# From Slow to Fast: Hypogravity-Induced Remodeling of Muscle Fiber Myosin Phenotype

**Published:** 2016

**Authors:** B. S. Shenkman

**Affiliations:** State Scientific Center of the Russian Federation – Institute of Biomedical Problems, Russian Academy of Sciences, Khoroshevskoe shosse, 76A, Moscow, 123007, Russia

**Keywords:** skeletal muscle, muscle fiber type, myosin heavy chain isoform, myosin phenotype, gravitational unloading, myosin gene expression

## Abstract

Skeletal muscle consists of different fiber types arranged in a mosaic pattern. These fiber types are characterized by specific functional properties. Slow-type fibers demonstrate a high level of fatigue resistance and prolonged contraction duration, but decreased maximum contraction force and velocity. Fast-type fibers demonstrate high contraction force and velocity, but profound fatigability. During the last decades, it has been discovered that all these properties are determined by the predominance of slow or fast myosin-heavy-chain (MyHC) isoforms. It was observed that gravitational unloading during space missions and simulated microgravity in ground-based experiments leads to the transformation of some slow-twitch muscle fibers into fast-twitch ones due to changes in the patterns of MyHC gene expression in the postural *soleus muscle*. The present review covers the facts and mechanistic speculations regarding myosin phenotype remodeling under conditions of gravitational unloading. The review considers the neuronal mechanisms of muscle fiber control and molecular mechanisms of regulation of myosin gene expression, such as inhibition of the calcineurin/NFATc1 signaling pathway, epigenomic changes, and the behavior of specific microRNAs. In the final portion of the review, we discuss the adaptive role of myosin phenotype transformations.

## INTRODUCTION. MYOSIN PHENOTYPE.

To the memory of K.B. Shapovalova together with whom the author studied the striopallidar control over the muscle myosin phenotype.

**Fig. 1:**
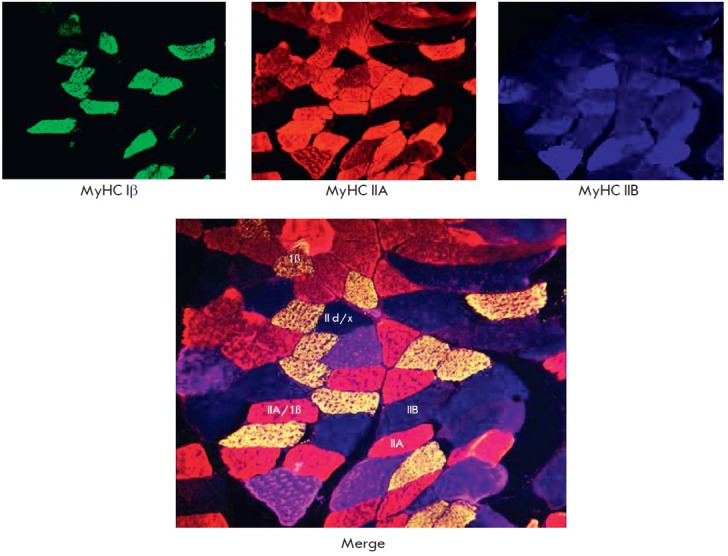
Immunocytochemical detection of muscle fibers expressing MyHC Iβ isoform, MyHC IIA, and MyHC IIB in the cross-section of *m. plantaris *of rats by the triplelabeling method. The main fiber types and hybrid fibers are shown.

Physiologists have investigated skeletal muscle fiber types since 1873 [1] the time it was established that muscles are composed of fibers with different functional properties and arranged in a mosaic pattern. Slow-twitch fibers are characterized by high fatigue resistance and a longer duration of contraction, but lower maximum force and velocity of contraction. Fasttwitch fibers are characterized by higher contraction velocity and force, but profound fatigability. In recent decades, it has been established that these properties are determined by the predominant isoform of the myosin heavy chain (MyHC). There are four isoforms, and, therefore, four types of fibers: I, slow; IIA, fast; IId/x fast; and IIB, the fastest one, which is represented only in the muscles of small mammals [2] *(Fig. 1, Table*). Myosin isoforms, prevailing in a fiber, determine its myosin phenotype, and the ratio of different types of fibers corresponds to muscle composition or the myosin phenotype. Along with fibers dominated by a certain type of MyHC isoform, muscles comprise fibers, having two (or more) different MyHC isoforms. These fibers are called hybrid fibers. The expression of each of the myosin isoforms is determined by fiber innervation. Fibers innervated by one motor neuron comprise a motor unit and, in the vast majority of cases, are characterized by the same myosin phenotype [3]. Postural (tonic) muscles, having a high tone and supporting the body’s posture in the Earth gravitational field, contain the largest amount of type I slow fibers. According to modern concepts, the motoneuron controls the fibers using a certain discharge frequency pattern (10 Hz for slow and 50–60 Hz for fast motor units) and secretion of the appropriate neurotrophic agents, which affects the expression of myosin genes: i.e. the myosin phenotype of the fibers [3, 4].

The myosin phenotype is very stable; however, there are impacts that can significantly alter the myosin gene expression and thereby determine the slow-to-fast transformation of fibers, or vice versa. For example, low-frequency electrostimulation during several weeks leads to the formation of 30–40% slow-type fibers in predominantly fast muscles [4]. The same effect in the fast ankle plantaris muscle was observed in animals with ablated or subjected to tenotomy triceps surae muscles: i.e. during the so-called compensatory overload [4]. In all these cases, the leading role in myosin phenotype transformations was attributed to changes in the muscle contractile activity pattern resulting from changes in the nature of the motor neuron discharge pattern (or, in the case of direct electrical stimulation, to its pattern).

**Table:** MyHC isoforms and muscle fiber types in mammals

MyHC isoform	β	α	Iβ	IIa	IId/x	IIb
Organ	Myocardium		MySkeletal muscleocardium			
Characteristics of species	All mammals					Small mammals
Contraction velocity	->					
Fatigue resistance	<-					

^#^The structure was solved by NMR in contrast to the other structures solved by X-ray crystallography.

## THE MECHANISMS OF ACTIVITY-DEPENDENT MYOSIN PHENOTYPE REMODELING

**Fig. 2:**
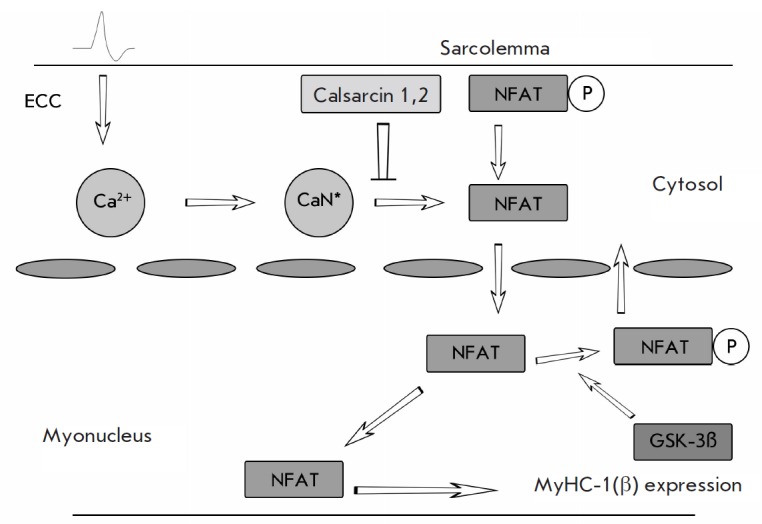
Functional diagram of the calcineurin/NFATc1 signaling pathway. (According to Liu et al. [16], revised). ECC – electromechanical coupling, CaN – calcineurin. Explanations are provided in the text.

**Fig. 3:**
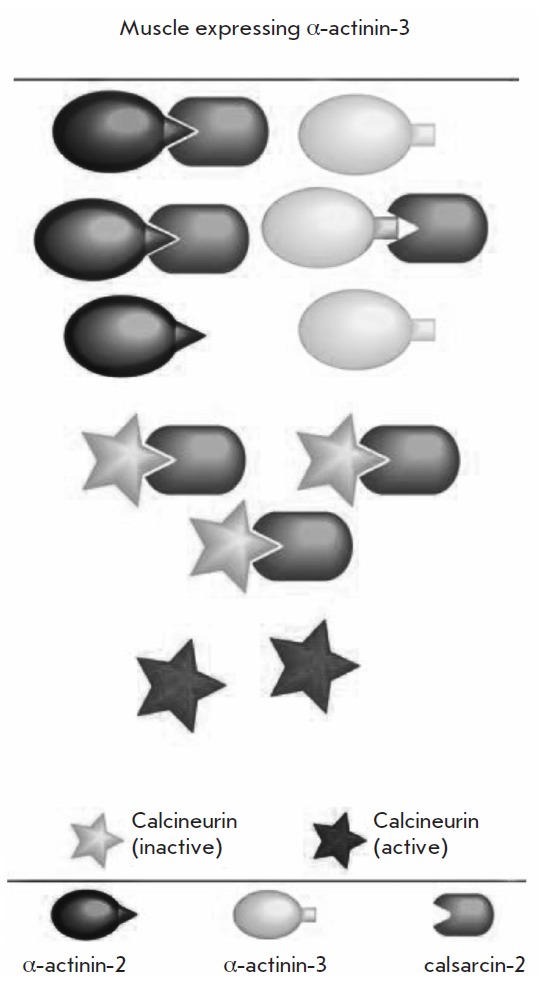
Calsarcin deposition diagram in α-actinin-2 and α-actinin-3 structures. (According to Seto et al. as revised in [13]). Explanations are provided in the text.

Chronic activity of slow-twitch fibers is associated with two phenomena: a constantly high myoplasm level of calcium ions and a low level of high-energy phosphates [4-6]. Therefore, the search for the signaling mechanisms that regulate MyHC gene expression was limited to identifying the pathways dependent on the concentration of calcium ions and high-energy phosphates. Calcineurin/NFAT is believed to be the most important signaling cascade that affects the expression of slow MyHC isoforms (and regulates the expression of many other genes). Calcineurin is a protein localized in the sarcomeric Z-disc. When interacting with the calcium- calmodulin complex, it displays phosphatase activity and dephosphorylates NFATs1 (the nuclear factor of activated T-cells), which can be translocated into myonuclei [6, 7] *(Fig. 2)*. In the nucleus, this factor is either stored in heterochromatin (and gradually transferred therefrom to euchromatin) [8] or directly interacts with MEF-2, a transcription factor specifically bound to the slow MyHC gene promoter. In this pattern, an intense transcription of slow the MyHC gene is initiated [7, 8]. The NFAT dephosphorylation reaction is inhibited by Z-disc proteins, calsarcin-1, and calsarcin-2, which operate in slow-twitch and fast-twitch fibers, respectively. Knockout of the genes of these proteins results in a significant redistribution of the myosin phenotype towards the slow type [9, 10] *(Fig. 2)*. Calsarcin gene expression (especially calsarcin-2) is inhibited in the case of double knockout of the E3 ubiquitin ligases MuRf- 1 and MuRf-2 [11]. It can be assumed that calsarcin-2 expression is stimulated by the presence of MuRf ubiquitin ligases in the nucleus. It has been shown that alteration of the titin/connectin state results in release/ dephosphorylation of MuRf-2 caused by the titin kinase domain localized on the M-disk, which leads to its import into myonuclei [12]. It is possible that titin alteration ultimately leads to increased expression of calsarcin-2, contributes to the stabilization of the fast myosin phenotype, and prevents any transformation towards the slow type. However, overexpression of the calsarcin gene is insufficient to completely inhibit the phosphatase activity of calcineurin. It is known that calsarcin-2 can be immobilized on the cytoskeletal components of Z-disc, α-actinin-2, and α-actinin-3, and immobilization on α-actinin-2 is more stable [13]. Therefore, in the absence of the α-actinin-3 gene or its deficit, calsarcin demonstrates stable immobilization and the slow-type phenotype of the fiber is produced* (Fig. *3).

Dephosphorylation of the GSK3β signaling protein (glycogen synthase kinase) promotes NFAT export from the nucleus and shifts the equilibrium toward the fast isoforms [14] *(Fig. 2)*. In this case, the GSK3β inhibitory activity may be suppressed by nitric oxide through the cGMP-pathway [15].

**Fig. 4:**
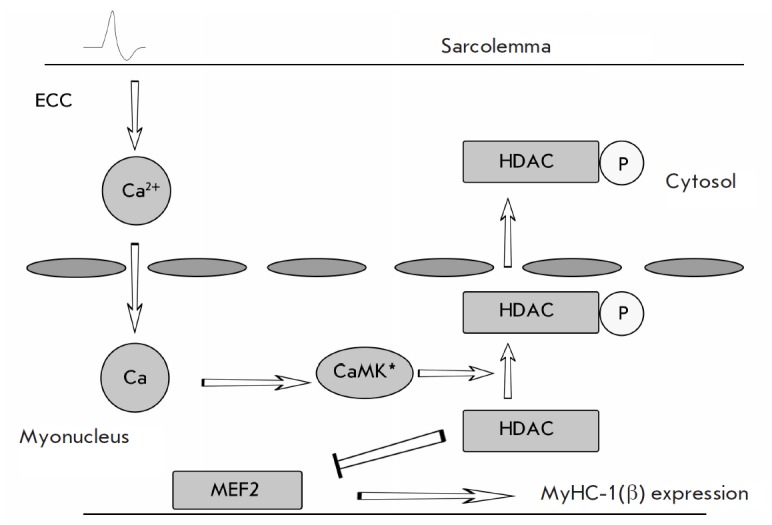
Functional diagram of the calcium-calmodulin kinase/histone deacetylase 4/5 signaling pathway (according to Liu et al. [17], revised). HDAC – histone deacetylase, CaMK – calcium-calmodulin kinase, MEF-2 – transcription factor (myocyte enhancement factor).

Another mechanism of myosin phenotype regulation ( also calcium-dependent) is implemented through the kinase activity of calcium-calmodulin kinase (CaMK). When activated by the calcium-calmodulin complex, this enzyme phosphorylates histone deacetylase 4 (HDAC4) and prevents it from entering the myonuclear space [16]. In the case of low concentration of the calcium-calmodulin complex and correspondingly low kinase activity of CaMK, HDAC4 is underphosphorylated and some of its molecules are translocated to myonuclei [17]. In myonuclei, HDAC4 deacetylates not only H3 histone, but also the MEF-2 transcription factor, which interacts with the *myf7 *gene promoter (i.e. MyHC Iβ gene) [17]. This leads to a decrease in the general transcriptional activity of the genome and expression of MyHC Iβ *(Fig. 4)*. Interestingly, here again, there is an “inhibiting” mechanism: HDAC4 can be ubiquitinylated and destroyed. This preserves the slow myosin phenotype [18].

The ratio of phosphorylated and non-phosphorylated high-energy phosphates, another physiological trigger of signaling processes, regulates the activity of AMP-dependent protein kinase (AMPK), which controls the main pathways of the energy metabolism of muscle fibers [19]. Additionally, AMPK phosphorylates the histone deacetylases HDAC4 and 5, which significantly facilitates the expression of the slow MyHC isoform and several other genes that control the regulatory proteins of oxidative metabolism [20, 21]. Furthermore, AMPK activity can be modulated (stimulated) by nitric oxide [22].

**Fig. 5:**
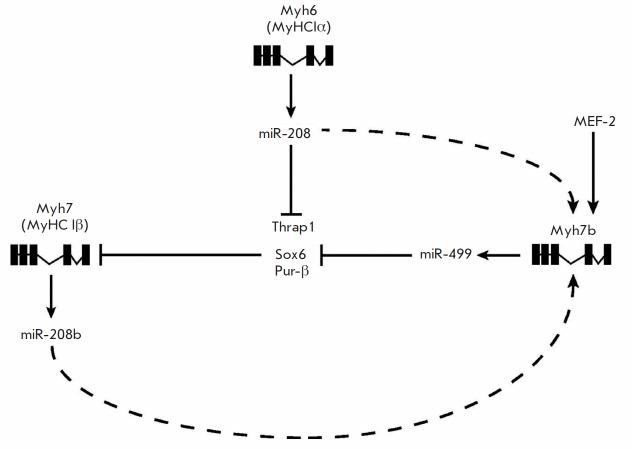
Participation of microRNA in the regulation of MyHC Iβ expression (according to McCarthy et al. [25]). Explanations are provided in the text.

Another mechanism of myosin phenotype modulation provides up-regulation of MyHC Iβ gene expression (*myh7 *gene*) *by means of microRNA. Besides the main MyHC Iβ gene (*myh7 *gene*), *mammalian genome comprises *myh7b (myh14) *gene, which is expressed in the skeletal muscles of adult mammalians in the form of mRNA; at the protein level, this gene is expressed only in the extraocular muscle [23]. However, its introns encode miR-499 microRNA. Expression of the *myh7b* gene is stimulated by miR-208b, which is encoded by the intron of *myh7, *the essential gene of slow myosin*.* In turn, miR-499 inhibits the expression of specific blockers of *myh7 *gene promoters (Sox6, Pur-β, and Thrap1) [24] *(Fig. 5)*. Interestingly, expression of the* myh7b *gene is stimulated by overexpression of MEF-2 (the basic transcriptional MyHC Iβ promoter) [25]. This suggests that an increase in the concentration of the calcium/calmodulin complex results in penetration of MEF-2, which can be dephosphorylated by calcineurin [26], to the nucleus, where it regulates *myh*7 expression. It also stimulates the synthesis of miR-499 that prevents the blockade of MyHC Iβ expression [25]. Thus, expression of miR-499 and miR-208b provides a smooth synthesis of slow myosin in the presence of an appropriate physiological stimulus (calcium ions).

## MYOSIN PHENOTYPE UNDER GRAVITATIONAL UNLOADING CONDITIONS

**Fig. 6:**
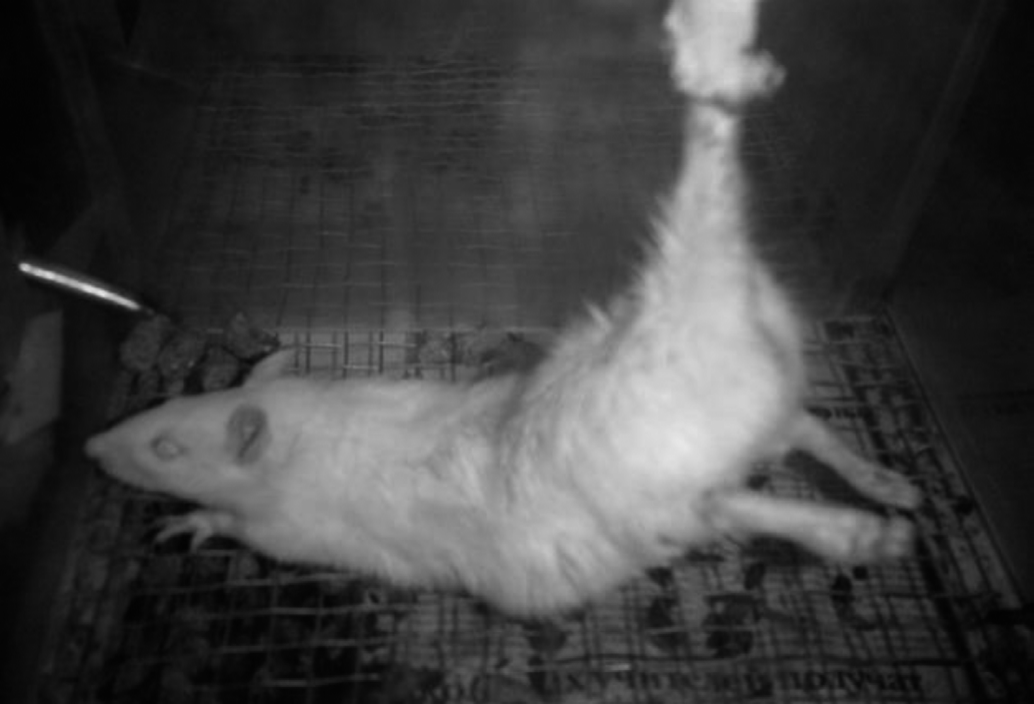
Il’in-Novikov method of rat suspension as revised by Morey-Holton.

Changes in the fiber myosin phenotype under gravitational unloading were observed in many laboratories. In particular, it was observed that rat hindlimb suspension results in increased content (%) of type II fibers and decreased proportion of type I fibers in *soleus muscle* [27-30] *(Fig. 6)*.

A seven-day spaceflight resulted in a slow-to-fast shift in the fiber type ratio in *soleus *and EDL *rat muscles* [31, 32]. In a 12.5- to 14-day flight, a decrease in the content of type I fibers in soleus and adductor longus muscles was observed [33, 34]. We were the first to discover an increased proportion of type II fibers in soleus and *vastus lateralis muscles *in monkeys after a 12.5-day spaceflight in the Kosmos-2229 biosatellite [35]. In cases when the shift in the fiber ratio could not be detected by staining for myofibrillar ATPase, an increased amount of fibers, reactive to fast myosin antibodies and a decreased amount of fibers reactive to slow myosin antibodies, was typically observed [36-41]. Electrophoresis revealed the emergence of a new isoform of myosin-heavy chains, 2d or 2x, in a suspension experiment [40]. An increased proportion of hybrid fibers consisting of both slow and fast forms of the myosin-heavy chain, was repeatedly detected in suspension experiments and spaceflights [37, 41]. A reduced proportion of fibers expressing the slow MyHC isoform and increased proportion of fibers expressing fast isoforms was also observed in *soleus muscle *samples from astronauts after a 6-month mission [42]. A shifted ratio of MyHC isoforms towards the fast type was detected using an electrophoretic analysis in the *vastus lateralis muscle *of astronauts after an 11-day flight [43]. In our lab, a reduced proportion of slow MyHC fibers in human soleus was observed as early as after a 7-day exposure to dry immersion [44, 45]. Interestingly, the intensity of the myosin phenotype transformation towards the slow type usually did not exceed 15–20% of the fibers, whereas other effects of muscle unloading involved most of the muscle fibers. This fact suggests that the final stabilization of the fast phenotype under unloading conditions is achieved only in part of the fibers being transformed.

## NEURONAL MECHANISMS OF MYOSIN PHENOTYPE REGULATION DURING GRAVITATIONAL UNLOADING

Several observations suggest that the elimination of support afferentation is the main mechanism leading to the “switching-off” of the electrical activity of postural muscle motor units during gravitational unloading (see review [44]). The use of mechanical stimulation of plantar support zones under these conditions maintains the normal level of electrical activity of postural muscles. Interestingly, the use of mechanical stimulation of plantar support zones during exposure to dry immersion enabled us to avoid a decrease in the proportion of slow fibers [44, 45]. When suspending rats with one hindlimb interacting with an artificial support, *the soleus muscle *of this leg demonstrated no myosin phenotype transformation towards the fast type, as opposed to the contralateral limb [46]. Low-frequency chronic electrostimulation of *rat soleus muscle *combined with the conventional suspension model also prevents myosin phenotype transformation [47, 48]. The same effects were observed after chronic muscle stretching or resistive exercises during gravitational unloading (suspension or 84-day bed rest) [49-51]. The results of these studies suggest that low-intensity muscular activity and resistive effects prevent changes in the myosin phenotype. Based on the aforementioned observations, we can suggest that the shift in myosin phenotype under gravitational unloading is caused, among other things, by changes in the neuronal control of motor unit activity. Indeed, the experiments with three-day dry immersion in humans revealed inactivation of slow-type motor units [52]. These results were confirmed in experiments with recording of the electrical activity of *soleus muscle *and fast synergists in *Macaca mulatta *during spaceflight [53] and rat hindlimb suspension, as well as their exposure under conditions of Kepler parabolic flight [54]. We can assume that it is the “switching-off” of slow motor units that leads to changes in the myosin phenotype in all of these cases. This hypothesis can be confirmed by the results obtained in the “spinal isolation” model, where all afferent and descending tracts to the lumbar spinal cord are dissected, while motor terminals are intact. In these experiments with complete “disconnection” of spinal motoneurons, myosin phenotype shift towards the fast type was observed [55]. When supplying chronic carbachol to striatopallidal structures during suspension, enhanced stability of the postural synergies in animals were even accompanied by an increase in the proportion of slow-type soleus fibers [56]. The disabling afferent activity of the tibialis anterior (TA) muscle (antagonist of *soleus muscle*) by means of tenotomy combined with hindlimb suspension prevents an increase in the proportion of fast-type fibers in murine *soleus muscle* [57]. It is conceivable that, during gravitational unloading, activation of the TA muscle [58] or the decrease in the intensity of the exciting striatopallidal effects [56] results in a decreased discharge activity of slow-type motor units of *soleus muscle *and, thus, leads to changes in the myosin phenotype of its fibers.

Another hypothetical neurophysiological mechanism of soleus motor unit inactivation under microgravity conditions is discussed in connection with the study of the muscle effects of vestibular deafferentation in animals. For this purpose, experiments with deafferentation of vestibular receptors using arsenilate injections were carried out [59]. After a month-long adaptation of rats to vestibular deafferentation, a decrease in the proportion of fibers expressing MyHC Iβ and their cross-sectional area, as well as an increase in the proportion of fibers expressing fast MyHC isoforms, was observed in *soleus muscle*. It is worthy of note that the discovered phenomenon is similar to the myosin phenotype transformation observed after spaceflights. This is indicative of the possibility that the functional changes in the vestibular apparatus in zero gravity state can contribute to changes in the nature of myosin isoform expression. This viewpoint is quite contestable. First, myosin phenotype transformation towards the fast type is also observed in ground-based zero gravity simulation models, when there is only mild alteration of the vestibular apparatus function (see above). Second, a similar study conducted using surgical vestibular deafferentation (labyrinthectomy) led to opposite changes in *soleus muscle *of animals. The myosin phenotype of *soleus muscle *shifted towards an increased proportion of slow-type fibers [60, 61]. Unfortunately, our knowledge of vestibular effects on the postural muscle myosin phenotype is limited to the aforementioned publications. Obviously, there remain many more questions than answers. Further research will contribute to filling in the blind spots in this field.

## EXPRESSION OF MYOSIN GENES UNDER CONDITIONS OF GRAVITATIONAL UNLOADING

**Fig. 7:**
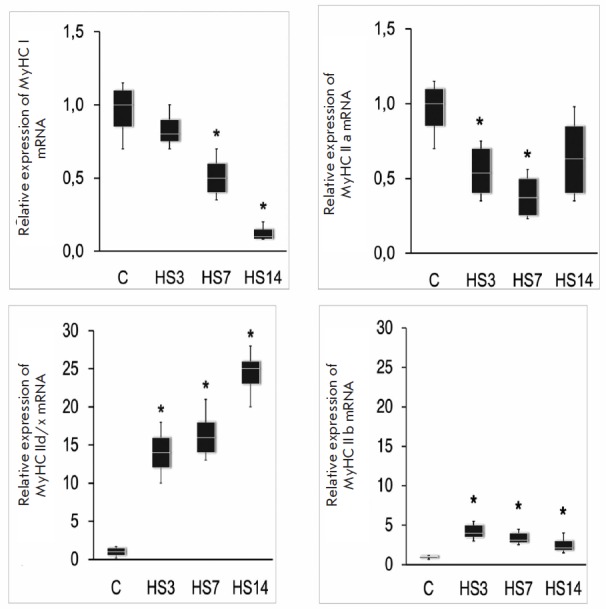
The dynamics of expression of MyHC isoform mRNA in rat *m. soleus* during unloading (suspension) [64]; HS3 – 3-day suspension, HS7 – 7-day suspension, HS14 – 14-day suspension. The data were obtained by quantitative real-time PCR.

At the beginning of this review, we stated that changes in the myosin phenotype during functional unloading (disuse) are determined by a decreased expression of the slow MyHC isoform gene and increased expression of the fast MyHC isoform gene ([4], etc.). It is interesting to follow the time-course dynamics of the process. Stevens et al. were the first to show that a mild decrease in the content of MyHC Iβ mRNA occurs as early as on the 4th day in suspended Wistar rats, and on the 7th day it becomes a trend and amounts to about 20% [62]. Researchers from the University of California, Irwin, detected a statistically significant decrease in the content of MyHC Iβ mRNA in Sprague-Dowley rats as early as after 24-hour suspension [63]. We observed a significant decrease in MyHC Iβ mRNA of Wistar rats on the 7th day of suspension, but a slight downward tendency was observed earlier, on the 3rd day [64] *(Fig. 7A)*. Thus, all these studies demonstrated a decrease in mRNA expression of the slow isoform of myosin heavy chains, but the speed of this process varied in different studies. Early and significant growth of the muscle content of mRNA encoding IIb and IId/x isoforms of myosin heavy chains *(Fig. 7C,D)* was also observed. Interestingly, after a 3- to 4-day suspension, there was not a single “pure” slow fiber in the pools of individual fibers: i.e. each fiber undergoes gradual replacement of MyHC Iβ by fast-type isoforms [65]. According to our data, the time-course dynamics of the MyHC IIA mRNA content [66] differs from the dynamics of MyHC Iβ mRNA, as well as MyHC IIb and IId/x mRNA. The content of MyHC IIA mRNA decreases after a 3-day suspension and further decreases up to day 7. After a 14-day suspension, the content of MyHC IIA mRNA was found to be so high that it did not differ from the control values *(Fig. 7B)*.

Thus, the changes in the myosin phenotype under gravitational unloading are preceded by changes in the expression pattern of mRNA encoding the corresponding MyHC isoforms. For this reason, the search for the molecular mechanisms of myosin phenotype transformation largely reduces to the study of the mechanisms of myosin gene expression regulation.

**Molecular regulatory mechanisms of gene expression of myosin heavy chain isoforms in postural muscles during unloading**

The mechanisms of the shift in the expression of MyHC isoform genes toward the fast type are still largely unexplored. The study of the role of the calcineurin/ NFATc1 signaling system during gravitational unloading revealed that intensive transportation of NFATc1 to the nuclei of rat soleus fibers [67] occurs after a 14- day suspension of Morey-Holton rats*. *However, the NFATc1 content in the myonuclei of human muscles is significantly reduced after a 60-day bed rest hypokinesia [68]. Obviously, there is a contradiction between these results. The issue of the intensity of the NFAT import to the nucleus during unloading remains unclear. Cyclosporin A, a NFATc1 dephosphorylation inhibitor [69, 70], was used in our laboratory and K.M. Baldwin’s laboratory to demonstrate that expression of slow-type MyHC mRNA is further reduced under the action of cyclosporin A, a calcineurin inhibitor, during suspension. This is indicative of the potential compensatory function of this signaling pathway during unloading. Furthermore, the difference between the intensity of the decrease in slow-type MyHC mRNA expression during unloading and under the same conditions, but with underlying administration of cyclosporin A, is small, but statistically significant. The similar amount of changes in this experiment indicate that downregulation of slowtype MyHC during unloading is largely due to inhibition of the calcineurin/NFATc1 signaling pathway.

**Fig. 8:**
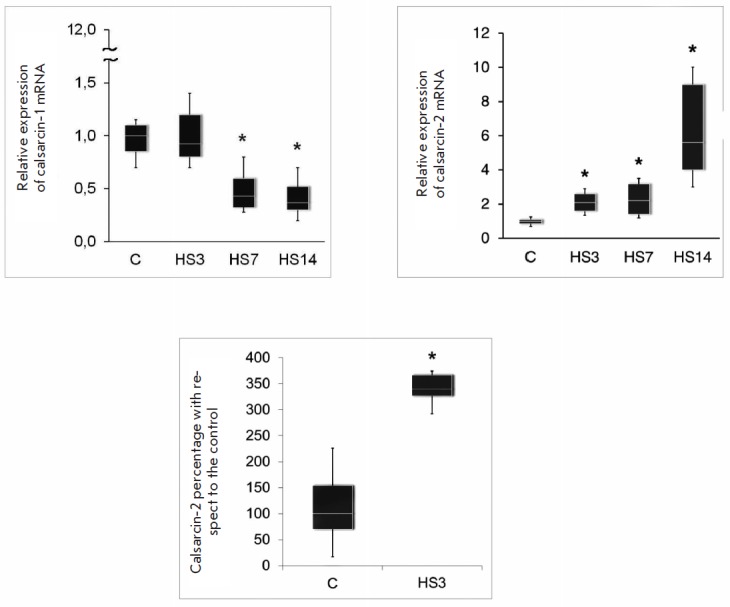
mRNA expression and the level of calsarcin proteins in rat *m. soleus *during unloading (suspension) [64]. HS3 – 3-day suspension, HS7 – 7-day suspension, HS14 – 14-day suspension. The data were obtained by quantitative real-time PCR and Western blotting (third diagram).

Transformation towards a fast phenotype does not occur when suspending mice knockout on both MuRF ubiquitin ligases [71]. Therefore, MuRF-dependent expression of calsarcin-2 is probably an important element for the stabilization of the fast myosin phenotype under the influence of hypothetical mechanisms whose compensatory effect is targeted at preserving a slow phenotype. We were the first to discover the isoform- specific time-course dynamics of calsarcin mRNA expression during simulated gravitational unloading *(Fig. 8)* [66]. On the 3rd day of suspension, the level of calsarcin-1 expression was the same as in the control, and then it decreased for up to 14 days. As early as on the 3rd day, the level of calsarcin-2 mRNA was twofold higher than in the control and it continued to increase up to day 14.

In view of both published and our own results, we can assume that, in the portion of fibers containing a significant proportion of fast MyHC isoforms, increased expression of calsarcin-2 results in the prevention of compensatory activation of the calcineurin pathway and, thereby, stabilization of the fast phenotype in these fibers. In other fibers (mostly slow ones), reduced calsarcin-1 expression may intensify the calcineurin pathway and, thereby, stabilize their slow phenotype. Thus, stable populations of slow and fast fibers with a significant shift towards the fast fiber type form by day 7. Additionally, we found a statistically significant increase in the level of MuRF-1 and MuRF-2 in the nuclear fraction of *rat soleus muscle *after a 3-day suspension; i.e., it is during this period that expression of calsarcin-2 increases [66]. This phenomenon, along with the effects of *murf *genes knockout [11], suggests the existence of a causal link between translocation of MuRF-1 and MuRF-2 to the nuclei at the initial stage of the unloading and increased calsarcin-2 expression.

It is possible that deposition of calsarcin in the structure of α-actinin-2 plays an important role in these processes. In our laboratory, a decreased content of α-actinin-2 in samples of murine *soleus muscle *was observed after a 7-day suspension of rats [72]. Therefore, it can be expected that bound calsarcin-2 is released due to alpha-actinin-2 degradation during simulated gravitational unloading. Cytoskeleton degradation during unloading is usually attributed to calcium-dependent cysteine proteases: calpains. It is, therefore, interesting that an increased expression of calpastatin, an endogenous calpain inhibitor, did not result in a transformation of the myosin phenotype towards the fast type in suspended mice [73]. The lack of transformation in these mice may be indicative of the fact that calpain activation can be one of the factors contributing to the transformation of the myosin phenotype during unloading. Since calpain activation during gravitational unloading is associated with the accumulation of calcium ions in the myoplasm [74-76], it is expected that blocking calcium ions delivery to the fiber when using nifedipine during gravitational unloading will result in decreased calpain activity and a less pronounced degradation of cytoskeletal proteins. Moreover, degradation of α-actinin-2 will not be as deep as in the case of suspension without additional action and the calsarcin depot will remain full. In this case, downregulation of MyHC Iβ will be completely or partially prevented. In support of this hypothesis, we found that there was no transformation of *rat soleus muscle *fibers in suspended rats administered chronic nifedipine [77]. However, the mechanisms of participation of calpains in MyHC expression regulation remain insufficiently studied.

In experiments on suspended rats in 2015, we observed activation (i.e., a decrease in the negative phosphorylation) of another endogenous inhibitor of the calcineurin/NFATc1 signaling pathway, glycogen synthase kinase GSK3β, which, in the absence of negative phosphorylation, phosphorylates NFATc1 and promotes its export from the nucleus [66]. The activity of this enzyme can be inhibited with a high content of nitrogen oxide in the fiber, which acts through the guanylate cyclase mechanism [78]. We have previously shown that the nitrogen oxide level in *rat soleus *is significantly reduced during gravitational unloading [79]. At the same time, administration of *L-*arginine, which enhances nitric oxide production, prevented a reduction of the MyHC Iβ mRNA content. Apparently, a decreased level of nitrogen oxide in the fiber during unloading can be considered as one of the stabilizing factors of the fast phenotype, which acts through GSK3β.

Salanova et al. [68] suggest that reduction in the intensity of NFATc1 import to myonuclei during functional unloading is associated with another mechanism: a decrease in Homer-1 scaffold protein expression, which was observed in human *soleus muscle *and *vastus lateralis muscle *after long-term bed rest hypokinesia. In that study, Homer-1 function is described as the scaffold support for approximation and interaction between calcineurin and NFATc1 in the postsynaptic area and Z-disc area. The mechanisms regulating the expression of this protein are not known.

The role of the ratio of high-energy phosphates in the control of the myosin phenotype under unloading conditions can be assessed only in the case when there is a significant change in this ratio at one or another stage of the process. Indeed, an early study of Ohira’s group revealed that a 10-day rat hindlimb suspension does increase the level of phosphocreatine in *rat soleus muscle *[80]. It turned out that a reduced level of phosphorylated high-energy phosphates due to administration of β-guanidinopropionic acid prevents the transformation of the myosin phenotype towards the fast type in suspended animals [81]. It is known that chronic administration of β-guanidinopropionic acid acts through AMPK-dependent signaling pathways [82]. Until recently, nobody knew how AMPK activity changed during unloading. The results of two studies in this field directly contradict each other [83, 84]. In our laboratory, it was shown that gravitational unloading using the conventional “dry” immersion model for 3 days results in a significant decrease in the AMPK phosphorylation level in human *soleus muscle *[85]. It is believed that phosphorylation/dephosphorylation of HDAC molecules is the main mechanism of AMPK impact on gene expression. It can be assumed that their action (deacetylation of H3 histone and MEF2 transcription factor) occurs during simulated gravitational unloading. Indeed, acetylation of H3 histone in the gene locus of the fast myosin isoform increases in suspended rats [86]. It was recently established that no slow-tofast fiber transformation occurs in *soleus muscle *of suspended rats subject to the action of the classical HDAC inhibitor [87].

The mechanism of microRNA-dependent regulation of myosin gene expression is also modulated under unloading conditions (see Introduction). Rat hindlimb suspension results in a reduced expression of miR-499 and miR-208b microRNA in the *soleus muscle*, and, therefore, there are conditions for the functioning of specific blockers of the *myh*7 gene promoter: i.e. reduced expression of slow myosin [25]. These data are consistent with the results of Tsika’ group demonstrating an increased expression of the blockers of the *myh*7 gene promoter, Pur-α, Pur-β, and SP3, and their binding to specific sites on the promoter during suspension [88, 89]. These processes may result from a reduced expression of the *myh7b *gene and miR-499. Little is known about the physiological regulators of specific blockers of *myh*7 gene expression and regulatory miR-499 and miR-208b.

The data on the regulation of *myh7 *gene expression provided in this review show that, despite the investigation of the molecular mechanisms that determine a reduced expression of slow MyHC isoforms under gravitational unloading, a complete picture of the functioning of these mechanisms cannot yet be built. It can be assumed that the functioning of a complex system of endogenous inhibitors of the calcineurin/NFATc1 signaling pathway is targeted at overcoming the compensatory muscle responses and fast phenotype stabilization. At the same time, it is unknown which epigenetic processes trigger the processes of *myh7 *gene inactivation and reduction of slow MyHC isoform expression at the very early stage of gravitational unloading during the first 24 hours.

Even less is known about the mechanisms that stimulate the functioning of the gene promoters of the fast MyHC isoform. It is believed that, in the absence of stimulants of the slow-type MyHC isoform, DNA binding to the MyoD transcriptional regulator enhances the expression of the fast-type myosin gene [90]. At the same time, MyoD knockout hindlimb unloaded animals demonstrate no transformation towards the fast type [91]. This fact suggests that MyoD significantly affects the expression of fast MyHC isoform genes during gravitational unloading. Interestingly, the stimulatory effect of MyoD on the expression of fast myosin isoforms is inhibited by NFATc1 [92]. Another reciprocal regulation mechanism is characteristic of the expression of MyHC IIA, on the one hand, and IId/x and IIb, on the other hand. It was found that spinal isolation results in a reduced expression of MyHC IIA and increased expression of IId/x [93]. We observed a similar phenomenon at the early stage of gravitational unloading in the experiments with hindlimb suspension [66]. It has been found that the MyHC IId/x gene promoter is located next to the MyHC IIA gene and transcription from the former occurs in two directions. Transcription from the sense strand triggers transcription of the IIx gene; antisense RNA is synthesized from the complementary strand, which leads to the destruction of MyHC IIA mRNA [93]. Thus, activation of the gene expression of the fast myosin isoform results in a reduced expression of the MyHC IIA gene.

## CONCLUSION

Regulation of myosin gene expression is being intensively studied at the moment. However, there is no clear picture of the long-known and still obscure phenomenon of the changing pattern of the expression of these genes during gravitational unloading. Basic questions concerning the described phenomenon will be answered in the near future. The adaptive role of the transformation of muscle fibers during gravitational unloading is not covered in numerous publications related to this problem. Hypogravity results in the “disabling” of mostly postural extensors, especially* soleus muscle*, and therein the fibers expressing the slow MyHC isoform and thus implementing slow “tonic” contractile activity. The changing nature of postural synergies under real and simulated zero gravity conditions leads to the elimination of the “tonic” component of the motor function. Therefore, the shift of the myosin phenotype towards the fast type can be an integral part of these adaptive rearrangements of the motor control system in mammals. Another view of the adaptive role of the myosin phenotype shift is based on the well-known differences in trophic mechanisms; i.e., the mechanisms that maintain the structure and metabolism of slow-type and fast-type muscle fibers. The elegant work of Ohira’s group [94] demonstrated that denervation of *rat soleus muscle,* combined with hindlimb suspension exposure, does not lead to an increase in atrophic changes, i.e. reduction of the fiber cross-sectional area. Under the same conditions, atrophy of the plantaris muscle was significantly less pronounced than atrophy of *soleus muscle,* but it was much more pronounced when the muscle was denervated. This is indicative of the fact that neurotrophic effects in the fast fiber effectively prevent the intensive development of atrophic processes. This strategy is not specific to slow-type fibers, whose structure is entirely determined by the intensity and duration of the contractile activity. It can be assumed that the transformation of the myosin phenotype of slow-type fibers changing them into fast-ones can increase the amount of fibers, preserving the volume of the myofibrillar apparatus during inactivity due to neurotrophic effects.

## References

[1] Ranvier L. (1873). CR Acad. Sci. Paris..

[2] Schiaffino S., Reggiani C. (2010). Physiol. Rev..

[3] Burke R.E. (1967). J. Physiol..

[4] Pette D. (2006). Skeletal muscle plasticity in health and disease / Eds Bottinelli R., Reggiani C. Springer, 2006.

[5] Tavi P., Westerblad H. (2011). J. Physiol..

[6] Chin E.R. (2010). Exerc. Sport Sci. Rev..

[7] Schiaffino S. (2010). Acta Physiol. (Oxf.)..

[8] Shen T., Liu Y., Contreras M., Hernández-Ochoa E.O., Randall W.R., Schneider M.F. (2010). Histochem. Cell Biol..

[9] Frey N., Frank D., Lippl S., Kuhn C., Kögler H., Barrientos T., Rohr C., Will R., Müller O.J., Weiler H., Bassel-Duby R., Katus H.A., Olson E.N. (2008). J. Clin. Invest..

[10] Frey N., Richardson J.A., Olson E.N. (2000). Proc. Natl. Acad. Sci. USA..

[11] Moriscot A., Baptista I.L., Bogomolovas J., Krohne C., Hirner S., Granzier H., Labeit S. (2010). J. Struct. Biol..

[12] Lange S., Xiang F., Yakovenko A., Vihola A., Hackman P., Rostkova E., Kristensen J., Brandmeier B., Franzen G., Hedberg B. (2005). Science..

[13] Seto J.T., Quinlan K.G., Lek M., Zheng X.F., Garton F., MacArthur D.G., Hogarth M.W., Houweling P.J., Gregorevic P., Turner N., Cooney G.J., Yang N., North K.N. (2013). J. Clin. Invest..

[14] Shen T., Cseresnyes Z., Liu Y., Randall W.R., Schneider M.F. (2007). J. Physiol..

[15] Martins K.J., St-Louis M., Murdoch G.K., MacLean I.M., McDonald P., Dixon W.T., Putman C.T., Michel R.N. (2012). J. Physiol..

[16] Liu Y., Shen T., Randall W.R., Schneider M.F. (2005). J. Muscle Res. Cell Motility..

[17] Liu Y., Randall W.R., Martin F., Schneider M.F. (2005). J. Cell Biol..

[18] Potthoff M.J., Wu H., Arnold M.A., Shelton J.M., Backs J., McAnally J., Richardson J.A., Bassel-Duby R., Olson E.N. (2007). J. Clin. Invest..

[19] Sanchez A.M., Candau R.B., Csibi A., Pagano A.F., Raibon A., Bernardi H. (2012). Amer. J. Physiol. Cell Physiol. 2012. V. 303. № 5. P. C475–485..

[20] Röckl K.S., Hirshman M.F., Brandauer J., Fujii N., Witters L.A., Goodyear L.J. (2007). Diabetes..

[21] McGee S.L., Hargreaves M. (2010). Clin. Sci. (London)..

[22] Lira V.A., Brown D.L., Lira A.K., Kavazis A.N., Soltow Q.A., Zeanah E.H., Criswell D.S. (2010). J. Physiol..

[23] Rossi A.C., Mammucari C., Argentini C., Reggiani C., Schiaffino S. (2010). J. Physiol..

[24] Van Rooij E., Quiat D., Johnson B.A., Sutherland L.B., Qi X., Richardson J.A., Kelm R.J.Jr., Olson E.N. (2009). Dev. Cell..

[25] McCarthy J.J., Esser K.A., Peterson C.A., Dupont-Versteegden E.E. (2009). Physiol. Genomics..

[26] Dunn S.E., Simard A.R., Bassel-Duby R., Williams R.S., Michel R.N. (2001). J. Biol. Chem..

[27] Templeton G.H., Sweeney H.L., Timson B.F., Padalino M., Dudenhoeffer G.A. (1988). J. Appl. Physiol..

[28] Desplanches D., Mayet M.H., Sempore B., Flandrois R. (1987). J. Appl. Physiol..

[29] Riley D.A., Slocum G.R., Bain J.L., Sedlak F.R., Sowa T.E., Mellender J.W. (1990). J. Appl. Physiol..

[30] Desplanches D., Kayar S.R., Sempore B., Flandrouis R., Hoppeler H. (1990). J. Appl. Physiol..

[31] Martin T.P., Edgerton V.R., Grindeland R.E. (1988). J. Appl. Physiol..

[32] Desplanches D., Mayet M.H., Ilyina-Kakueva E.I., Sempore B., Flandrois R. (1990). J. Appl. Physiol..

[33] Desplanches D., Mayet M.H., Ilyina-Kakueva E.I., Frutoso J., Flandrois R. (1991). Eur. J. Appl. Physiol..

[34] Miu B., Martin T.P., Roy R.R., Oganov V.S., Ilyina-Kakueva E.I., Marini J.F., Leger J.J., Bodine-Fowler S., Edgerton V.R. (1990). FASEB J..

[35] Shenkman B.S., Kozlovskaya I.B., Kuznetsov S.L., Nemirovskaya T.L., Desplanches D. (1994). J. Gravit. Physiol. 1994. V. 1. № 1. P. P64–.

[36] Baldwin K.M., Herrick R., Ilyina-Kakueva E.I., Oganov V.S. (1990). FASEB J..

[37] Ohira Y., Jiang B., Roy R.R., Oganov V., Ilyina-Kakueva E., Marini J.F., Edgerton V.R. (1992). J. Appl. Physiol..

[38] Guezennec C.Y., Gilson E., Serrurier B. (1990). Eur. J. Appl. Physiol..

[39] Campione M., Ausoni S., Guezennec C., Shiaffino S. (1993). J. Appl. Physiol..

[40] Takahashi H., Wada M., Katsuta S. (1991). Acta Physiol. Scand..

[41] Thomason D., Morrison P.R., Oganov V., Ilyina-Kakueva E.I., Booth F.W., Baldwin K.M. (1992). J. Appl. Physiol..

[42] Trappe S., Costill D., Gallagher P., Creer A., Peters J.R., Evans H., Riley D.A., Fitts R.H. (2009). J. Appl. Physiol..

[43] Zhou M.Y., Klitgaard H., Saltin B., Roy R.R., Edgerton V.R., Gollnick P.D. (1995). J. Appl. Physiol..

[44] Grigor’ev A.I., Kozlovskaia I.B., Shenkman B.S. (2004). The role of support afferents in organisation of the tonic muscle system. Ross Fiziol Zh Im I M Sechenova..

[45] Shenkman B.S., Podlubnaia Z.A., Vikhliantsev I.M., Litvinova K.S., Udal’tsov S.N., Nemirovskaia T.L., Lemesheva Iu.S., Mukhina A.M., Kozlovskaia I.B. (2004). Human soleus fibers contractile characteristics and sarcomeric cytoskeletal proteins after gravitational unloading. Contribution of support stimulus. Biofizika..

[46] Nemirovskaya T.L., Shenkman B.S. (2002). Eur. J. Appl. Physiol..

[47] Leterme D., Falempin M. (1994). Pflug. Arch..

[48] Dupont E., Cieniewski-Bernard C., Bastide B., Stevens L. (2011). Am. J. Physiol. Regul. Integr. Comp. Physiol..

[49] Falempin M., Mounier Y. (1998). Acta Astronautics. 1998. V. 42. № l–8..

[50] Podlubnaia Z.A., Vikhliantsev I.M., Mukhina A.M., Nemirovskaia T.L., Shenkman B.S. (2004). Sarcomeric cytoskeletal proteins and myosin phenotype in stretched soleus of hindlimb- suspended rats. Biofizika..

[51] Gallagher P., Trappe S., Harber M., Creer A., Mazzetti S., Trappe T., Alkner B., Tesch P. (2005). Acta Physiol. Scand..

[52] Kirenskaia A.V., Kozlovskaia I.B., Sirota M.G. (1986). Effect of immersion hypokinesia on the characteristics of the rhythmic activity of the motor units of the soleus muscle. Fiziol Cheloveka..

[53] Roy R.R., Hodgson J.A., Aragon J., Day M.K., Kozlovskaya I., Edgerton V.R. (1996). J. Gravit. Physiol..

[54] Kawano F., Nomura T., Ishihara A., Nonaka I., Ohira Y. (2002). Neurosci..

[55] Huey K.A., Roy R.R., Baldwin K.M., Edgerton V.R. (2001). Muscle Nerve..

[56] Shenkman B.S., Shapovalova K.B., Mukhina A.M., Kozlovskaia I.B., Nemirovskaia T.L., Kamkina Iu.V. (2006). Activation of neostriatum muscarinic receptors prevents changes in the myosin phenotype of musculus soleus fibers under gravitational unloading.. Dokl Biol Sci..

[57] Shenkman B.S., Nemirovskaya T.L., Mukhina A.M., Podlubnnaya Z.A., Vikhlyantsev I.M., Ardabyevskaya A.V., Kozlovskaya I.B., Grigoriev A.I. (2005). Effects of inactivation of the antagonist muscle on the atrophic processes in rat soleus muscle under conditions of gravitational unloading.. Dokl. Akad. Nauk..

[58] Yuganov E.M., Kasyan I.I., Tcherepakhin M.A., Gorshkov A.I. (1962). On some human responses under conditions of reduced weight-bearing. Probl. Kosm. Biol..

[59] Luxa N., Salanova M., Schiffl G., Gutsmann M., Besnard S., Denise P., Clarke A., Blottner D. (2013). J. Vestib. Res..

[60] Fuller Ch. (2002). XII Conf. on space biology and aerospace medicine, Moscow..

[61] Kasri M., Picquet F., Falempin M. (2004). Exp. Neurol..

[62] Stevens L., Sultan K.R., Peuker H., Gohlsch B., Mounier Y., Pette D. (1999). Am. J. Physiol. Cell Physiol..

[63] Giger J.M., Bodell P.W., Zeng M., Baldwin K.M., Haddad F. (2009). J. Appl. Physiol..

[64] Shenkman B.S., Lomonosova Y.N. (2014). Expression of calsarcin isoforms and myosin phenotype stabilization in transitional unloaded muscle.. Dokl Biochem Biophys..

[65] Stevens L., Gohlsch B., Mounier Y., Pette D. (1999). FEBS Lett..

[66] Lomonosova Y.N., Turtikova O.V., Shenkman B.S. (2016). J. Muscle Res. Cell Motility..

[67] Dupont-Versteegden E.E., Knox M., Gurley C.M., Houle J.D., Peterson C.A. (2002). Am. J. Physiol. Cell Physiol..

[68] Salanova M., Bortoloso E., Schiffl G., Gutsmann M., Belavy´ D.L., Felsenberg D., Sandra Furlan S., Volpe P., Blottner D. (2011). FASEB J..

[69] Lomonosova Y.N., Shenkman B.S., Nemirovskaya T.L. (2009). Calcineurin-mediated regulation of myosin heavy chain expression in rat soleus muscle under conditions of reduced motor activity. Ross Fiziol Zh Im I M Sechenova..

[70] Pandorf C.E., Jiang W.H., Qin A.X., Bodell P.W., Baldwin K.M., Haddad F. (2009). Am. J. Physiol. Regul. Integr. Comp. Physiol..

[71] Labeit S., Kohl C.H., Witt C.C., Labeit D., Jung J., Granzier H. (2010). J. Biomed. Biotechnol..

[72] Mirzoev TM., Shenkman BS., Ushakov IB., Ogneva I.V. (2012). Desmin and α-actinin-2 content in rat soleus muscle in the dynamics of gravitational unloading and subsequent reloading. Dokl Biochem Biophys..

[73] Tidball J.G.., Spencer M.J. (2002). J. Physiol..

[74] Ingalls C.P., Warren G.L., Armstrong R.B. (1999). J. Appl. Physiol..

[75] Ingalls C.P., Wenke J.C., Armstrong R.B. (2001). Aviat. Space Environ. Med..

[76] Kandarian S.C., Stevenson E.J. (2002). Exerc. Sport Sci. Rev..

[77] Mukhina A.M., Altaeva E.G., Nemirovskaia T.L., Shenkman B.S. (2006). Role of L-type Ca channels in Ca2+ accumulation and changes in distribution of myosin heavy chain and SERCA isoforms in rat M. soleus under gravitational unloading. Ross Fiziol Zh Im I M Sechenova..

[78] Drenning J.A., Lira V.A., Simmons C.G., Soltow Q.A., Sellman J.E., Criswell D.S. (2008). Am. J. Physiol. Cell Physiol..

[79] Lomonosova Y.N., Kalamkarov G.R., Bugrova A.E., Shevchenko T.F., Kartashkina N.L., Lysenko E.A., Shvets V.I., Nemirovskaya T.L. (2012). Protective effect of L-Arginine administration on proteins of unloaded m. soleus.. Biochemistry (Mosc)..

[80] Wakatsuki T., Ohira Y., Yasui W., Nakamura K., Asakura T., Ohno H., Yamamoto M. (1994). Jpn. J. Physiol..

[81] Matoba T., Wakastuki T., Ohira Y. (1993). Med. Sci. Sports Exerc..

[82] Zong H., Ren J.M., Young L.H., Pypaert M., Mu J., Birnbaum M.J., Shulman G.I. (2002). Proc. Natl. Acad. Sci. USA..

[83] Han B., Zhu M.J., Ma C., Du M. (2007). Appl. Physiol. Nutr. Metab..

[84] Hilder T.L., Baer L.A., Fuller P.M., Fuller C.A., Grindeland R.E., Wade C.E., Graves L.M. (2005). J. Appl. Physiol..

[85] Vilchinskaya N.A., Mirzoev T.M., Lomonosova Y.N., Kozlovskaya I.B., Shenkman B.S. (2015). J. Musculoskelet. Neuronal Interact..

[86] Pandorf C.E., Haddad F., Wright C., Bodell P.W., Baldwin K.M. (2009). Am. J. Physiol. Cell. Physiol..

[87] Dupré-Aucouturier S., Castells J., Freyssenet D., Desplanches D. (2015). J. Appl. Physiol..

[88] Tsika G., Ji J., Tsika R. (2004). Mol. Cell. Biol..

[89] Ji J., Tsika G.L., Rindt H., Schreiber K.L., McCarthy J.J., Kelm R.J. Jr., Tsika R. (2007). Mol. Cell. Biol.

[90] Wheeler M.T., Snyder E.C., Patterson M.N., Swoap S.J. (1999). Am. J. Physiol..

[91] Seward D.J., Haney J.C., Rudnicki M.A., Swoap S.J. (2001). Am. J. Physiol. Cell. Physiol..

[92] Ehlers M.L., Celona B., Black B.L. (2014). Cell Rep..

[93] Pandorf C.E., Haddad F., Roy R.R., Qin A.X., Edgerton V.R., Baldwin K.M. (2006). J. Biol. Chem..

[94] Ohira Y., Yoshinaga T., Ohara M., Kawano F., Wang X.D., Higo Y., Terada M., Matsuoka Y., Roy R.R., Edgerton V.R. (2006). Cells Tissues Organs..

